# *catena*-Poly[[bromidocopper(I)]-μ-η^2^,σ^1^-3-(2-allyl-2*H*-tetra­zol-5-yl)pyridine]

**DOI:** 10.1107/S1600536808010313

**Published:** 2008-05-03

**Authors:** Wei Wang

**Affiliations:** a Ordered Matter Science Research Center, Southeast University, Nanjing 210096, People’s Republic of China

## Abstract

The title compound, [CuBr(C_9_H_9_N_5_)]_*n*_, has been prepared by the solvothermal treatment of CuBr with 3-(2-allyl-2*H*-tetra­zol-5-yl)pyridine. It is a new homometallic Cu^I^ olefin coord­ination polymer in which the Cu^I^ atoms are linked by the 3-(2-allyl-2*H*-tetra­zol-5-yl)pyridine ligands and bonded to one terminal Br atom each. The organic ligand acts as a bidentate ligand connecting two neighboring Cu centers through the N atom of the pyridine ring and the double bond of the allyl group. A three-dimensional structure is formed through weak Cu—Br [3.1579 (8) Å], C—H⋯Br and C—H⋯N inter­actions.

## Related literature

For the solvothermal synthesis and related structures, see: Ye *et al.* (2005 ▶, 2007 ▶).
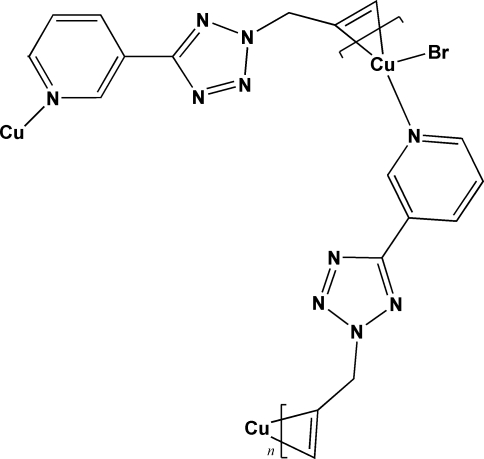


## Experimental

### 

#### Crystal data

[CuBr(C_9_H_9_N_5_)]
*M* *_r_* = 330.66
Triclinic, 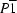

*a* = 7.4464 (15) Å
*b* = 7.7982 (16) Å
*c* = 9.940 (2) Å
α = 80.15 (3)°
β = 76.02 (3)°
γ = 85.13 (3)°
*V* = 551.3 (2) Å^3^
*Z* = 2
Mo *K*α radiation
μ = 5.58 mm^−1^
*T* = 293 (2) K
0.2 × 0.15 × 0.1 mm

#### Data collection

Rigaku Mercury2 diffractometer
Absorption correction: multi-scan (*CrystalClear*; Rigaku, 2005 ▶) *T*_min_ = 0.720, *T*_max_ = 1 (expected range = 0.412–0.572)
5748 measured reflections
2522 independent reflections
2173 reflections with *I* > 2σ(*I*)
*R*_int_ = 0.032

#### Refinement

*R*[*F* ^2^ > 2σ(*F* ^2^)] = 0.032
*wR*(*F* ^2^) = 0.072
*S* = 1.11
2522 reflections
145 parameters
H-atom parameters constrained
Δρ_max_ = 0.41 e Å^−3^
Δρ_min_ = −0.44 e Å^−3^

### 

Data collection: *CrystalClear* (Rigaku, 2005 ▶); cell refinement: *CrystalClear*; data reduction: *CrystalClear*; program(s) used to solve structure: *SHELXS97* (Sheldrick, 2008 ▶); program(s) used to refine structure: *SHELXL97* (Sheldrick, 2008 ▶); molecular graphics: *PLATON* (Spek, 2003 ▶) and *CAMERON* (Pearce *et al.*, 2000 ▶); software used to prepare material for publication: *SHELXL97*.

## Supplementary Material

Crystal structure: contains datablocks I, global. DOI: 10.1107/S1600536808010313/dn2335sup1.cif

Structure factors: contains datablocks I. DOI: 10.1107/S1600536808010313/dn2335Isup2.hkl

Additional supplementary materials:  crystallographic information; 3D view; checkCIF report

## Figures and Tables

**Table 1:** Hydrogen-bond geometry (Å, °)

*D*—H⋯*A*	*D*—H	H⋯*A*	*D*⋯*A*	*D*—H⋯*A*
C1—H1⋯Br1^i^	0.93	2.90	3.776 (3)	157
C2—H2⋯N5^i^	0.93	2.62	3.415 (4)	144
C9—H9*A*⋯N3^ii^	0.97	2.88	3.800 (4)	159

Symmetry codes: (i) 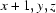; (ii) 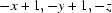.

**Fig. 1.**
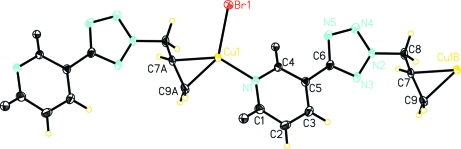
A view of the title compound with the atomic-labelling scheme. Displacement ellipsoids are drawn at the 30% probability level. [Symmetry codes: (i) x,y + 1, z - 1; (ii) x, y - 1, z + 1].

**Fig. 2.**
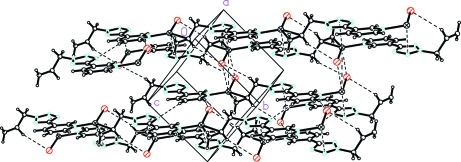
Partial packing view of the title compound showing the formationof the three dimensionnal network. Weak interactions are shown as dashed lines. H atoms not involved in hydrogen bondings have been omitted for clarity.

## supplementary crystallographic information

### Comment

Under hydrothermal or solvothermal conditions, some interesting reactions can be carried out leading to new compounds, while It is worth noting that these products could not be synthesized using conventional solution techniques. In sealed tube, unstable copper (I) salt can exist under vacuums, and then interesting copper (I) coordination compound can be obtained (Ye *et al.*, 2005, 2007). The title compound is obtained through solvothermal treatment of CuBr and 3-(2-allyl-2H-tetrazol -5-yl) pyridine in methanol solvent at 75°C, colorless block crystals suitable for X-ray diffractions have been isolated. have been isolated.

The copper(I) is coordinated to two olefinic ligands and one terminal Br atom in a trigonal environment (Fig 1). The olefin ligands link the neighbouring Cu centers to form an homometallic Cu^I^ coordination polymer developping along the *c* axis. The 3-(2-allyl-2*H*-tetrazol-5-yl) pyridine ligands coordinate to copper (I) centers through N atom of pyridine ring and double bond of allyl group. Unfortunately, the N atoms of tetrazole ring fail to coordinate to Cu(I).

Finally, weak Cu—Br, C-H···Br and C—H···N (Table 1) interactions result in the formation of a three-dimensionnal structure (Fig. 2)

### Experimental

A mixture of 3-(2-allyl-2H-tetrazol-5-yl) pyridine(20 mg, 0.2 mmol), CuBr (17.9 mg,0.2 mmol), and methanol (2 mL) sealed in a glass tube were maintained at 75 °C. Crystals suitable for X-ray analysis were obtained after 5 days

### Refinement

All H atoms were fixed geometrically and treated as riding with C—H = 0.93 Å (aromatic), 0.97 Å (methylene) or 0.96Å (methyl) with *U*_iso_(H) = 1.2*U*_eq_(Caromatic, Cmethylene) or *U*_iso_(H) = 1.5*U*_eq_(Cmethyl).

### Crystal data

**Table:**

[CuBr(C_9_H_9_N_5_)]	*Z* = 2
*M**_r_* = 330.66	*F*(000) = 324
Triclinic, *P*1	*D*_x_ = 1.992 Mg m^−^^3^
Hall symbol: -P 1	Mo *K*α radiation, λ = 0.71073 Å
*a* = 7.4464 (15) Å	Cell parameters from 5524 reflections
*b* = 7.7982 (16) Å	θ = 3.1–28.8°
*c* = 9.940 (2) Å	µ = 5.58 mm^−^^1^
α = 80.15 (3)°	*T* = 293 K
β = 76.02 (3)°	Block, colorless
γ = 85.13 (3)°	0.2 × 0.15 × 0.1 mm
*V* = 551.3 (2) Å^3^	

### Data collection

**Table:**

Rigaku Mercury2 diffractometer	2522 independent reflections
Radiation source: fine-focus sealed tube	2173 reflections with *I* > 2σ(*I*)
graphite	*R*_int_ = 0.032
Detector resolution: 13.6612 pixels mm^-1^	θ_max_ = 27.5°, θ_min_ = 3.1°
CCD_Profile_fitting scans	*h* = −9→9
Absorption correction: multi-scan (*CrystalClear*; Rigaku, 2005)	*k* = −10→10
*T*_min_ = 0.720, *T*_max_ = 1	*l* = −12→12
5748 measured reflections	

### Refinement

**Table:**

Refinement on *F*^2^	Primary atom site location: structure-invariant direct methods
Least-squares matrix: full	Secondary atom site location: difference Fourier map
*R*[*F*^2^ > 2σ(*F*^2^)] = 0.032	Hydrogen site location: inferred from neighbouring sites
*wR*(*F*^2^) = 0.072	H-atom parameters constrained
*S* = 1.11	*w* = 1/[σ^2^(*F*_o_^2^) + (0.0241*P*)^2^ + 0.2889*P*] where *P* = (*F*_o_^2^ + 2*F*_c_^2^)/3
2522 reflections	(Δ/σ)_max_ = 0.001
145 parameters	Δρ_max_ = 0.41 e Å^−^^3^
0 restraints	Δρ_min_ = −0.44 e Å^−^^3^

### Special details

**Table:**

Geometry. All esds (except the esd in the dihedral angle between two l.s. planes) are estimated using the full covariance matrix. The cell esds are taken into account individually in the estimation of esds in distances, angles and torsion angles; correlations between esds in cell parameters are only used when they are defined by crystal symmetry. An approximate (isotropic) treatment of cell esds is used for estimating esds involving l.s. planes.
Refinement. Refinement of *F*^2^ against ALL reflections. The weighted *R*-factor *wR* and goodness of fit *S* are based on *F*^2^, conventional *R*-factors *R* are based on *F*, with *F* set to zero for negative *F*^2^. The threshold expression of *F*^2^ > σ(*F*^2^) is used only for calculating *R*-factors(gt) *etc*. and is not relevant to the choice of reflections for refinement. *R*-factors based on *F*^2^ are statistically about twice as large as those based on *F*, and *R*- factors based on ALL data will be even larger.

### Fractional atomic coordinates and isotropic or equivalent isotropic displacement parameters (Å^2^)

**Table:**

	*x*	*y*	*z*	*U*_iso_*/*U*_eq_	
Br1	−0.16126 (4)	0.38319 (4)	0.41251 (3)	0.03175 (10)	
Cu1	0.16354 (5)	0.33917 (5)	0.39002 (4)	0.03347 (12)	
N1	0.2917 (3)	0.5093 (3)	0.2281 (2)	0.0267 (5)	
N2	0.0895 (4)	0.9916 (3)	−0.2237 (2)	0.0269 (5)	
N3	0.2398 (4)	0.9414 (3)	−0.1753 (2)	0.0282 (5)	
N4	−0.0584 (4)	0.9032 (4)	−0.1582 (3)	0.0357 (6)	
N5	−0.0058 (4)	0.7876 (4)	−0.0601 (3)	0.0344 (6)	
C1	0.4652 (4)	0.5523 (4)	0.2192 (3)	0.0346 (7)	
H1	0.5264	0.4971	0.2872	0.042*	
C2	0.5561 (5)	0.6741 (5)	0.1144 (4)	0.0380 (8)	
H2	0.6777	0.6985	0.1099	0.046*	
C3	0.4632 (4)	0.7608 (4)	0.0144 (3)	0.0325 (7)	
H3	0.5213	0.8450	−0.0573	0.039*	
C4	0.2039 (4)	0.5909 (4)	0.1310 (3)	0.0251 (6)	
H4	0.0844	0.5603	0.1355	0.030*	
C5	0.2839 (4)	0.7195 (4)	0.0238 (3)	0.0244 (6)	
C6	0.1752 (4)	0.8133 (4)	−0.0724 (3)	0.0256 (6)	
C8	0.0820 (5)	1.1435 (4)	−0.3331 (3)	0.0311 (7)	
H8A	0.1252	1.2437	−0.3063	0.037*	
H8B	−0.0457	1.1694	−0.3395	0.037*	
C7	0.1968 (4)	0.1148 (4)	0.5261 (3)	0.0275 (6)	
H7	0.1777	0.0060	0.4956	0.033*	
C9	0.3637 (4)	0.1859 (4)	0.4666 (4)	0.0377 (8)	
H9A	0.4502	0.1221	0.4010	0.045*	
H9B	0.4217	0.2348	0.5280	0.045*	

### Atomic displacement parameters (Å^2^)

**Table:**

	*U*^11^	*U*^22^	*U*^33^	*U*^12^	*U*^13^	*U*^23^
Br1	0.02503 (17)	0.03096 (17)	0.03858 (18)	−0.00123 (12)	−0.01088 (13)	0.00150 (13)
Cu1	0.0248 (2)	0.0368 (2)	0.0298 (2)	0.00004 (16)	−0.00487 (16)	0.01707 (17)
N1	0.0230 (13)	0.0300 (13)	0.0224 (12)	0.0013 (10)	−0.0036 (10)	0.0059 (10)
N2	0.0302 (13)	0.0260 (12)	0.0209 (11)	−0.0013 (10)	−0.0060 (10)	0.0063 (10)
N3	0.0328 (14)	0.0269 (13)	0.0206 (11)	−0.0037 (10)	−0.0046 (10)	0.0065 (10)
N4	0.0316 (15)	0.0371 (15)	0.0316 (14)	−0.0044 (12)	−0.0035 (12)	0.0096 (12)
N5	0.0296 (14)	0.0376 (15)	0.0295 (13)	−0.0074 (11)	−0.0056 (11)	0.0140 (12)
C1	0.0295 (17)	0.0415 (18)	0.0306 (16)	−0.0001 (14)	−0.0104 (13)	0.0048 (14)
C2	0.0269 (17)	0.0453 (19)	0.0396 (18)	−0.0110 (14)	−0.0080 (14)	0.0048 (15)
C3	0.0337 (17)	0.0300 (16)	0.0296 (15)	−0.0072 (13)	−0.0048 (13)	0.0065 (13)
C4	0.0240 (15)	0.0265 (14)	0.0209 (13)	−0.0017 (12)	−0.0028 (11)	0.0040 (11)
C5	0.0273 (15)	0.0246 (14)	0.0187 (13)	−0.0004 (12)	−0.0033 (11)	0.0006 (11)
C6	0.0307 (16)	0.0242 (14)	0.0185 (13)	−0.0028 (12)	−0.0020 (12)	0.0014 (11)
C8	0.0387 (18)	0.0250 (15)	0.0246 (14)	0.0021 (13)	−0.0069 (13)	0.0075 (12)
C7	0.0307 (16)	0.0217 (14)	0.0258 (14)	0.0031 (12)	−0.0078 (12)	0.0075 (12)
C9	0.0286 (17)	0.0375 (18)	0.0389 (18)	0.0074 (14)	−0.0084 (14)	0.0130 (15)

### Geometric parameters (Å, °)

**Table:**

Br1—Cu1	2.3752 (7)	C2—H2	0.9300
Cu1—N1	2.001 (2)	C3—C5	1.377 (4)
Cu1—C9	2.040 (3)	C3—H3	0.9300
Cu1—C7	2.057 (3)	C4—C5	1.387 (4)
N1—C4	1.337 (3)	C4—H4	0.9300
N1—C1	1.341 (4)	C5—C6	1.463 (4)
N2—N4	1.320 (3)	C8—C7^i^	1.495 (4)
N2—N3	1.326 (3)	C8—H8A	0.9700
N2—C8	1.472 (3)	C8—H8B	0.9700
N3—C6	1.330 (4)	C7—C9	1.361 (4)
N4—N5	1.320 (4)	C7—C8^ii^	1.495 (4)
N5—C6	1.353 (4)	C7—H7	0.9800
C1—C2	1.370 (4)	C9—H9A	0.9700
C1—H1	0.9300	C9—H9B	0.9700
C2—C3	1.395 (4)		
			
N1—Cu1—C9	107.38 (12)	C5—C4—H4	118.7
N1—Cu1—C7	145.02 (11)	C3—C5—C4	118.6 (3)
C9—Cu1—C7	38.80 (12)	C3—C5—C6	121.4 (3)
N1—Cu1—Br1	108.50 (7)	C4—C5—C6	119.9 (3)
C9—Cu1—Br1	144.01 (9)	N3—C6—N5	112.7 (3)
C7—Cu1—Br1	105.36 (9)	N3—C6—C5	123.6 (3)
C4—N1—C1	118.2 (3)	N5—C6—C5	123.5 (3)
C4—N1—Cu1	121.3 (2)	N2—C8—C7^i^	112.7 (2)
C1—N1—Cu1	120.37 (19)	N2—C8—H8A	109.1
N4—N2—N3	114.7 (2)	C7^i^—C8—H8A	109.1
N4—N2—C8	122.0 (3)	N2—C8—H8B	109.1
N3—N2—C8	123.1 (2)	C7^i^—C8—H8B	109.1
N2—N3—C6	101.0 (2)	H8A—C8—H8B	107.8
N2—N4—N5	105.8 (2)	C9—C7—C8^ii^	123.7 (3)
N4—N5—C6	105.9 (2)	C9—C7—Cu1	69.93 (17)
N1—C1—C2	122.8 (3)	C8^ii^—C7—Cu1	106.21 (19)
N1—C1—H1	118.6	C9—C7—H7	115.6
C2—C1—H1	118.6	C8^ii^—C7—H7	115.6
C1—C2—C3	118.9 (3)	Cu1—C7—H7	115.6
C1—C2—H2	120.6	C7—C9—Cu1	71.26 (18)
C3—C2—H2	120.6	C7—C9—H9A	116.5
C5—C3—C2	118.8 (3)	Cu1—C9—H9A	116.5
C5—C3—H3	120.6	C7—C9—H9B	116.5
C2—C3—H3	120.6	Cu1—C9—H9B	116.5
N1—C4—C5	122.7 (3)	H9A—C9—H9B	113.5
N1—C4—H4	118.7		

Symmetry codes: (i) *x*, *y*+1, *z*−1; (ii) *x*, *y*−1, *z*+1.

### Hydrogen-bond geometry (Å, °)

**Table:**

*D*—H···*A*	*D*—H	H···*A*	*D*···*A*	*D*—H···*A*
C1—H1···Br1^iii^	0.93	2.90	3.776 (3)	157.
C2—H2···N5^iii^	0.93	2.62	3.415 (4)	144.
C9—H9A···N3^iv^	0.97	2.88	3.800 (4)	159.

Symmetry codes: (iii) *x*+1, *y*, *z*; (iv) −*x*+1, −*y*+1, −*z*.
